# The New Local Anæsthetic—Hydrochlorate of Cocaine

**Published:** 1885-01

**Authors:** J. Edw. Line


ARTICLE VI.

### THE NEW LOCAL ANÆSTHETIC—COCAINE HYDROCHLORATE.

BY J. EDW. LINE, D. D. S.

[Read before the Rochester Dental Club, Nov. 11th, 1884.]

The comparatively recent statements in regard to this drug as a local anæstnetic, the present impossibility of obtaining a grain of it even for experimental purposes, and its very high price, make it an object of rare interest to every one, and especially to the dentist—not, however, because of what is known of it as a dental therapeutic so much as what is suspected and expected of it as an aid in our work upon sensitive organs and tissues.

As its name suggests, it is the product resulting from the treatment of cocaine with chlorhydric acid. Cocaine is the alkaloid obtained from the *Cuca* of the South American Indians, or *Coca* of the Spaniards, and coca is a plant of the natural order Erythroxylaceæ, resembles the black thorn, and grows to a height of six or eight feet. The leaves are used as a masticatory in the western countries of South America, and are the parts in which is found the alkaloid,

"to which cuca owes its special properties." Good specimens of the dried leaves "have a strong tea-like order; when chewed they produce a sense of warmth in the mouth, and have a pleasant, pungent taste. Bad specimens have a camphoraceous smell, * * * and lack the pungent taste." The leaves are used by the South American as generously and as industriously as prepared tobacco is by persons nearer home—the figures being placed at 8,900,000, and the chewers in Bolivia, Peru, Ecuador, Columbia, and Rio Negro.

Cuca is said to be "a powerful stimulant of the nervous system; it enables tatigue fatigue to be borne with less nourishment and greater ease than ordinarily, and diminishes the difficulty of breathing in ascending mountains." It is also said to be "a remedy for rheumatism and headache." Poppig, in his *Travels in China and Peru*, says the cuca-chewing habit is "as dangerous to health as opium-eating;" and Lloyd in the *Journal of the Royal Geographical Society* (1854,) speaks of it as a "poisonous narcotic." Other observing travellers and experimenters, however, testify to the contrary, and seem to commend rather than condemn the leaf and its use by human beings. The best kind of argument against Popping and Lloyd's statements is found in the number who habitually use the stuff, and the further fact that an able bodied South American Indian easily and habitually gets away with from two to three ounces in a day.

Cocaine ($C_{16}H_{19}NO_4$) is an alkaloid of *Erythroxylon Coca*, and the substance to which *cuca*, as already stated, owes its special properties. It was discovered in '59 by Niemann. "It is highly poisonous, and its physiological action is apparently identical with that of theine, cafeine, guaranine, and therobromine, which all (as shown by Bettet in the *EdinburghMedicalJournal*, '73) * * * induce a series of symptoms affecting the nervous, respiratory, circulatory, vasa-motor, and glandular systems."

The muriate of cocaine, or cocaine hydrochlorate, is found mentioned in Merck's catalogue. It had been known a year or more to laryngologists as an excellent application to the vocal cords, its anæsthetic effect facilitating examinations. It was the observance of this fact that led young Koller of Vienna to experiment with it upon the eye, and it was he who supplied Brettauer with the vial of the solution used by him in eye operations before the Ophthalmological Congress at Heidelberg last September.

According to Dr. H. D. Noyes, of New York, one of the special correspondents of the *Medical Record*, it was the most notable thing presented on that occasion. He says: "Two drops of the solution (a two *per cent.*) were dropped into the eye of the patient at the first experiment, and after an interval of ten minutes it was evident that the sensitiveness of the surface was below the normal, then two drops more were instilled and after waiting ten minutes longer there was entire absence of senstbility ; a probe was pressed upon the cornea until its surface was indented, it was rubbed lightly over the surface of the cornea, it was rubbed over the conjunctiva bulbi, and of the conjunctiva palpebrarum ; a speculum was introduced to separate the lids and they were stretched apart to the uttermost ; the conjunctiva bulbi was seized by the fixation forceps and the globe moved in various directions. In all this handling the patient declared that he felt no unpleasant sensation, except that the speculum stretched the lids so widely asunder as to give a little discomfort at the outer canthus. Before the experiment his eye was showd to possess the normal sensitiveness, and the other eye, which was not experimented on, was in this respect perfectly normal. The solution caused no irritation of any kind, nor din it at all influence the pupil. The anæsthetic influence seemed to be complete on the surface of the eye, and it lasted for about fifteen minutes, and the parts then resumed their usual condition."

Immediately upon the publication of Dr. Noyes' letter, specialists sought the dealer, the major part of them only to learn that the whole stock had been gathered in by earlier comers. A few of the more fortunate ones have already been enabled to put the drug to the test and publish their results. Prof. C. P. Agnew and Drs. William O. Moore and James S. Minor, who devote themselves to the treatment of the eye and ear. corroborate all that Dr. Noyes has said, and add new cases and new interest to the history of this most welcome agent. Dr. Agnew, in a case of convergent squins in a five year old, cut the scleral conjunctiva and internal rectus muscle; also the same muscle in a eleven year old, and in both without causing pain. Another case (six years of age) of the same kind failed because of the "nervous apprehension" of the child. Examinations only were made in two cases, in one of which the parts were sensitive to a remarkable degree to both torch and light—a case of laceration of the eye-ball, involving the sclerotic. The other was a casc of double cataract—sensitive, of course, to touch only. Dr. Moore scraped an ulcer in a Chinaman's cornea, freely cut the conjunctiva in another patient, bringing the cut surfaces together by three sutures, rnd in a child of seven cut the internal rectus. Dr. Minor removed stitches from the conjunctiva of a ten year old boy, put there a few days before under other and less pleasant circumstances; and in a case of cataract, in a woman of fifty, made a large section of the cornea, and accomplished cystotomy and the removal of the lens easily and painlessly. All of which shows that cocaine hydrochlorate is prompt in its action, and effective even at some distance from the surfaces to which it is applied.

Dr. Geo. F. Shrady says: "It is quite certain that in those numerous cases in which local anæsthesia is necessary for minor operations in surgery, gynecology, laryngology, otology, and even dentistry, this anæsthetic will be tested. Especially would it seem to be indicated in loose parts of the body which are covered by mucus membranes and plentifully supplied with sensitive nerves." A week ago last Wednesday, Prof. W. M. Polk, of N. Y., performed painless operations upon two women for laceration of the neck of the uterus. He is now engaged in experimenting with the drug to determine its effects when applied to the cervix and vagina in the exceedingly distressing pains of the first stage of labor.

Our own interest in cocaine hydrochlorate dates from the 20th of October, when we read the reports of Agnew, Moore and Minor. In our innocence we applied to a local drug firm for some of it, and was informed that they were not only wiihout it, but did not know what it was. It was found in their copy Merck's catalogue as the alkaloid only, and worth $4.00 per 15 grains. This firm wrote immediately to Merck's agents in New York, and two days later got for answer: "Have cabled for it."

As a consequence we are obliged to suggest a few of its probable applications in dentistry, instead of giving, as we had hoped when we said we would furnish a topic for this evening, the results of a series of experiments.

In tooth-extraction it may not be efficient, except perhaps to obtund the sensitiveness of the parts to which the beaks of the forceps are applied. As a remedy for tooth-ache, whether from an exposed pulp, or one whose decomposition has been interfered with, it promises well. And from what has been done by the oculists we may rest assured of its effectiveness in cases where the use of the lance is called for, as preliminary to tooth-extraction, envacuation of abscesses, scarrification of the gums, and the removal of abnormal growths. It seems, too, that it must work satisfactorily as an application to the gums previous to the introduction of the peg-wood wedge. But of all things calling most loudly for suspension of sensation, dentine ranks first, and if cocaine hydrochlorate meets this want, it will supply one much as well as long felt.—*Odontographic Journal.*

